# Taking emic and etic to the family level: interlinking parents’ and children’s COVID-19 views and experiences in Germany

**DOI:** 10.1186/s12889-024-18983-z

**Published:** 2024-06-17

**Authors:** Astrid Berner-Rodoreda, Nina Baum, Jonas Wachinger, Kathrin Zangerl, Henriette Hoegl, Till Bärnighausen

**Affiliations:** 1 Heidelberg Institute of Global Health, University of Heidelberg, INF 130.3, 69120 Heidelberg, Germany; 2 Kindernetzwerk e.V, Am Glockenturm 6, 63814 Mainaschaff, Germany

**Keywords:** emic, etic, children, parents, COVID-19

## Abstract

**Background:**

COVID-19 impacted families globally, restricting movement, and changing daily routines and family dynamics. In order to explore and contrast children’s and parents’ experiences and perceptions of life during COVID-19, we used Pike’s distinction of emic (an insider’s view) and etic (an outsider’s view) and adapted the concept to the family level to differentiate between children’s and parents’ own perspectives (emic) and their view of other family members (etic).

**Methods:**

Our qualitative study is based on face-to-face in-depth individual interviews with parents (*n* = 13) and their children (*n* = 16) and included migrant families as a hitherto underrepresented group in COVID-19 research in Germany. Interviews were recorded, transcribed in NVivo and quality-checked. We employed thematic analysis to explore similarities and differences in perceptions and experiences of children and parents at the family level and across the entire data set.

**Results:**

We identified the following major themes in parents’ and children’s experiences: managing role and relationship changes within the nuclear family, coping with social expectations and demands, and re-evaluations of life’s priorities. Parents’ etic views on children showed strong overlap with children’s emic view in terms of physical movement restrictions, experiencing good and tense family times, and internalizing rules. For issues such as experiencing stigma, divorce or language acquisition, parents’ views were not reflected in children’s accounts. Children’s testing experience, by contrast, was more nuanced than parents’ perceptions of it. Children’s etic views of parents, a perspective rarely found in qualitative research with children, overlapped with mothers’ experiences of role strain.

**Conclusions:**

The consideration of parents’ and children’s emic and etic perspectives provided deeper insights into family members’ experiences, navigation, and views of COVID-19 measures. Applying the emic/etic distinction to the family context enriches the sociology of childhood studies and enables a more nuanced understanding of diverging experiences within families and should thus be further explored within and beyond epidemics in order to guide future pandemic measures.

## Introduction

### COVID-19 affecting families

SARS-CoV-2 and precautionary measures to curb its spread have impacted families around the world in multiple ways and to varying degrees. Children had to cope with lockdowns, school closures, home-schooling and restrictions in seeing friends and relatives [1, 2], and families had to adapt to disruptions of their daily lives and routines [3]. A meta-analysis of systematic reviews highlighted a high burden of isolation-related mental health problems [4], with higher intensity levels of stress being associated with younger people and women [5, 6]. Studies in various countries showed children’s quality of life suffering in terms of less optimism, increased stress and decreased physical activity [7]. During COVID-19, children, adolescents and adults spent more time in front of screens [8] exacerbating already existing problems of inertia among adolescents [9].

Studies have also highlighted parents’ role strain during COVID-19 [9, 10]. Having to take on a teaching role during home-schooling and shifting the workplace to home made it harder for parents, particularly mothers, to achieve a work-life balance [11], as they were exposed to increased stress, and had to find mechanisms to cope [12]. Our interest in this study is to explore COVID-19 perspectives and experiences of parents and children and their views of each other (see following sections), rather than pursuing a family systems approach [13, 14] that hones in on child development and family functioning through family relationships.

### Emic and etic perspectives applied to studies with children

The linguist Pike coined the terms *emic* and *etic* “to describe behavior from two different standpoints” [15]. Emic denotes the perspective from “within a system” (idem) and etic from “outside of a particular system” (idem). The terms became widely used in anthropology [16] with emic pertaining to meaningfulness in the eyes of actors themselves and etic to their observable behaviour (idem). They have been applied in diverse fields and have been linked to a discussion about which research approach might be more adequate and where to position the researcher in this dichotomy [17, 18]. Generally, it is understood as an insider (emic) versus an outsider (etic) perspective [19, 20]. We are applying the terminology here in a more granular way: to understand parents’ and children’s own experiences from their own point of view (emic) and from the other group’s point of view (etic; e.g., parents commenting on children’s behaviour or experiences and vice versa).

Approaches to studying children and childhood changed over the course of the 20th century as Moran-Ellis noted: “the dominance of both developmental psychology and socialisation led to an almost exclusive focus in the social sciences on mapping and marking children’s progress towards adult functioning and societal membership as adults” [21]. From the 1980/90s, rather than studying how children were formed by families and the wider society, sociological childhood studies started to focus on children’s own agency, and research *with* rather than *about* children subsequently increased. This aligns with direct participation of children in matters that concern them as laid down in the Convention of the Rights of the Child [22]. Instead of collecting parents’ and teachers’ narratives about children’s experiences (which, in our usage of the terms, would be an etic approach), studies increasingly focus on asking children about their own experiences [23–25]. Children’s own (emic) experiences have been termed a *children’s* perspective, parents’ or teachers’ (etic) perspectives of children a *child* perspective in sociological studies on children [26, 27]. Children’s (etic) perceptions of their parent(s)’ experiences seem less prominent in qualitative studies.

### Parents’ and children’s perspectives of COVID-19

COVID-19 studies employing what we term an emic approach or a *children’s* perspective [27] underscore positive feelings about family quality time [25, 28] and staying in touch with remote relatives virtually [25], but also depict missing friends, outings, and celebrations as negative [25, 29] and home-schooling as mixed experiences [23, 30]. Differing etic perceptions of parents, in some cases teachers and children were highlighted with regard to online schooling [31] and physical activities [32]. Studies also provided nuances regarding school transitions, which were felt to be an incisive experience by all participants [33]. Parents employing a *child* or etic perspective noted greater anxiety in their children [34], which was echoed in another study by children who emphasized their fear for others’ safety and wellbeing [28].

Emic and etic views can converge [15], also in the family context where children might be influenced by their parents‘ views [26], resulting in a spectrum of potential alignment of children’s and parents’ perspectives to stark divergence. Speaking to children and parents as different stakeholder groups, i.e. practicing “data triangulation” [35], enriches the understanding of “lived experiences” [36], as parents’ (etic) views add another dimension and additional aspects to the (emic) experience reported by children [37].

Within-family differences of COVID-19 experiences and attitudes have been explored in few studies so far. Qualitative studies with migrant parents and children underscored the challenges of online learning, educational inequalities and language acquisition [38, 39], yet did not compare and contrast perspectives.

To explore emic and etic perspectives within the family context, we incorporate parents’ and children’s own experience as well as *child* and *parent* views (etic perceptions of the experience of the respective other). This approach is in line with Pike’s recommendation to differentiate standpoints [15], but we take a more granular emic/etic perspective of the microcosm of family life in this qualitative COVID-19 study in Germany: we apply the emic/etic distinction not primarily to the perspective of the researcher vis-à-vis interviewees but to different interviewees’ perspectives.

## Methods

Between May and September 2022, we conducted qualitative research with families, who had lived in the state of Baden-Württemberg, Germany, through the various COVID-19 waves.

### Study setting and COVID-19 situation

The south-western state of Baden-Württemberg is the third largest state in Germany in terms of population size, geographic area, and the absolute number of foreigners residing in the state.^1^

In Germany, COVID-19 related restrictions varied according to the epidemic wave, the state and area one lived in. Five COVID-19 waves preceded the time of the interviews. Wave 1 lasted from March-May 2020, wave 2 from September 2020-February 2021, wave 3 from February 2021-May/June 2021, wave 4 from July-December 2021, and wave 5 from December 2021-May 2022 [40]. Baden-Württemberg imposed school and kindergarten closures during wave 1 with emergency schooling offered for parents in jobs relevant to the system. Playgrounds were cordoned off, mask-wearing was compulsory in public places, and restrictions on socializing applied. In June 2020, all schools re-opened with an alternate schooling concept that could differ from school to school. Measures became intensified (mask-wearing in schools) with more social restrictions from October to December that turned into a hard lockdown in December with schools closing until March 2021. Teachers could then get vaccinated, and demonstrations against COVID-19 measures started. In April 2021, only exam students returned to school. For others, alternate schooling was practiced with compulsory COVID-19 tests – previously infected or vaccinated students were exempted. Curfews were imposed. In May 2021, swimming pools and restaurants opened with restrictions, linking measures to incidence. In September, students had to test three times per week; in December, mask-wearing became compulsory again due to the new omicron variant. In March 2022, mask-wearing and distancing at schools stopped.^2^

### Sampling and data collection

We purposively sampled families with one or more children aged 6-17^3^ who had lived in Germany since the beginning of 2020, the global and German onset of COVID-19. The aim was to reach a heterogenous sample of families including migrant families from diverse socio-cultural and economic backgrounds to (a) learn from the rich experience of families in Germany with children growing up bilingually and feeling at home in more than one culture, and (b) to include families that are often underrepresented in research [41] but emerged as an important group affected by COVID-19 measures [5]. The first and second author established contact with eligible families on the recommendations of research colleagues and their social networks. In most cases, mothers were the first point of contact and facilitated study participation of other family members.

Following voluntary written consent (see *Ethics approval*), the first and second author conducted all interviews face-to-face using precautions such as wearing face-masks. The interviews were mostly conducted at the family’s home, in a room of the participant’s or parent’s choosing. One family preferred a public space for convenience. While we conducted individual interviews, parents and children could decide on the presence of a parent during the child’s interview [42]. In most families, a parent was present or within earshot for at least one of the children interviewed; in one family, the parent preferred to be in sight but could not hear the interview with the child. Visiting families at home allowed for witnessing the interactions of family members and contextualizing the interviews through observations, an additional important method in qualitative research [43].

For interviewing parents, we drew on a narrative interview style [44], asking them to recall what life was like before COVID-19 and to take us through the last three years of their lives. This qualitative interview method allowed parents to set their own narrative focus before asking them more pointedly about their experiences or perceptions [44]. In order to make it less awkward for children to talk about their COVID-19 experiences to a stranger, the first two authors asked them to imagine the interviewer as an aunt who lived far-away, could not visit them during COVID-19, and was curious about the past three years of their life. For children (6–9 yrs) and adolescents (10 years plus), who found it challenging to remember the time before COVID-19 and to go through the various changes they had experienced, we shifted to a semi-structured interview. We asked them about their daily routine before COVID-19 and during the COVID-19 precautionary measures with follow-up questions on schooling/home-schooling, leisure activities, friendships and family relationships. Their COVID-19 experiences of proximity and distance and drawings, furnished as part of the interview, have been published elsewhere [45].

Interviews were either conducted in German or English according to the participant’s preference and, in the case of the parents of a West Asian family, with Arabic translation. The audio-recorded interviews were transcribed verbatim and quality-checked. Interview duration ranged from 16 to 69 min.

### Analysis

After conducting the interviews, the two lead authors shared their observations, interview experience and interview contents in order to debrief other team members and learn from each interview [46]. A reflexive thematic analysis approach was employed [47–50]. The first two authors transcribed all interviews: author 2 transcribed most interviews; author 1 checked all transcripts against the audio. Interview transcripts were read several times to deepen familiarization with the data [47]. The first two authors summarized the interviews independently, compared these to enrich each other’s understanding and shared the summaries with the wider team. Manual coding in NVivo Pro 12 was employed to further deepen familiarization with the data [47]. While the first author inductively coded all interviews, the second author independently coded a subset thereof. Coding was informed by analysing the transcripts, not by questions from the interview guide. Discussing the coding deepened our understanding of the data – we had no interest in establishing coder reliability which we view as a misguided concept in a constructivist approach [50]. In a next step, the first two authors independently extracted and juxtaposed the experiences of children and parents at family level according to themes identified through the coding. They discussed and compared their individual tables and produced an enriched version for the wider research team. Family tables were transferred into a matrix to provide an overview of the entire data-set. Overlapping and diverging experiences and perspectives of participants were discussed in the wider team, and issues and themes identified that affected predominantly one group (such as role strains and the cementing of gender roles) or both (such as coping with social demands), see below.

### Reflexivity

The first and second author have a background in social anthropology. The first author is a middle-aged German woman, who holds a PhD in public health, has worked within and outside of Europe and is married to a non-German. Working as a global consultant for many years exposed her to different cultural settings so that interviewing people of diverse ages and backgrounds felt comfortable. In addition to conducting qualitative research on communicable diseases, she also became infected in the first wave of COVID-19, and experienced COVID-19 measures when procedures were not yet in place in Germany. This family study on COVID-19 combined professional and personal interests to explore how families coped with the pandemic and its social restrictions.

The 2nd author is a German woman in her 20s who grew up in a family with a non-migratory background. She was therefore younger than the parents she interviewed but closer in age to the adolescent children. At the time of research, she had not been infected with COVID-19. She is presently finalizing her Master’s Degree for which she spent time outside of Europe for data collection, familiarizing herself with different cultural settings.

While both authors were affected by COVID-19, neither of them had to look after school children during the pandemic.

## Results

The first two authors conducted 29 in-depth interviews with members of nine families. In seven out of the nine families, at least one parent had a migratory background, see Table 1. Families will be referred to in the order they were interviewed:

**Table 1:** List of families

Family	Region of origin (of at least one parent)	Household members	No. of interviewees
1	Western Europe (no migratory background)	6	4
2	Western Europe	5	3
3	Western Europe	5	3
4	West Asia	2	2
5	Eastern Europe	3	3
6	Western Europe (no migratory background)	6	4
7	East Asia	3	3
8	West Asia	4	4
9	West Africa	6	3

In order to protect the families we will only mention regions and not countries. Two families had one child; other families had 2–5 children, see Table 1. No more than two children were interviewed per family. For information on interviewees’ age and gender, see Table 2.

**Table 2:** Socio-demographic data

Interview Participants	Female (*n*)	Male (*n*)	Mean Age (SD*)	Age Range
Parents	9	4	41.0 (3.65)	35–48
Children	5	4	7.9 (1.05)	6–9
Adolescents	4	3	13.0 (1.83)	10–15

*Standard Deviation

### A matter of perspective

Although parents and their children were generally exposed to the same COVID-19 restrictions and precautionary measures (e.g. lockdowns, school closures), the felt impact was verbalized differently. Our research team identified the following themes: Managing role and relationship changes within the nuclear family, coping with social expectations and demands, and re-evaluating restrictions and life’s priorities. These will be addressed one by one, highlighting differences and similarities in the views of parents and children.

#### Managing role and relationship changes within the nuclear family

Participants highlighted the profound impact of COVID-19 on their families. On the one hand, lockdowns led to uncertainties for many families who were not sure what the coming months had in store and how well they would handle the situation financially; on the other hand, life slowed down for everyone. More time was spent within the nuclear family, and children did not have to be chaperoned to their leisure activities. Spending more time together as a family meant quality time and establishing new family routines, yet, particularly for mothers, lockdowns and home-schooling caused additional stress. Some interviewees highlighted the cementing of gendered roles and more family tensions as social interactions were predominantly restricted to the nuclear family. This will be elaborated on in the following sub-sections.

##### Parents dealing with role strains

Parents’ initial relief of having fewer or no external work engagements and leisure-time activities for the children soon gave way to being burdened with additional roles and responsibilities at home, ranging from becoming the main playmate to instructing children in school subjects and organizing their lives: “And then, I only realized what changes this brought with it regarding our role, that we were suddenly everything as parents. Well, mainly me […] All of a sudden, I was occupied with restructuring everyday life for the children” (Family 1, Mother). Home-schooling led to additional tasks for mothers: assisting the children with online-schooling, printing or collecting homework assignments, and uploading them. Organizing everyday life was perceived as “even more than the usual multi-tasking […] Home-schooling and working at the same time is impossible… It’s really just like going over the limit of what you can handle with three kids” (Family 3, Mother). Having to juggle several parental roles was further exacerbated by the need to help children with school work that they struggled with on their own as expressed by one of the children.

I found the exercises difficult to do and then, the teacher did not explain things and that made it even more difficult, because I had to always go to my mum or see by myself how to deal with it. (Family 6, Son, 9 yrs)

Parental support in school assignments was a salient issue for both children and parents as remarked upon by a father: “I felt that teachers confused them [the children] a bit with university students who can work independently” (Family 1, Father). He further explained how parents had to constantly check on the school’s online system to ensure that their children had completed all their schoolwork. Fathers also viewed their roles as having multiplied: helping with household chores or setting up the computer, checking the equipment, testing the children for COVID-19 before school, supporting the child with schoolwork, or accompanying the child to their sport or music leisure activities when this was possible. “Well, I had to be a parent, husband, pedagogue, ehm, primary school teacher, tutor, playmate” (Family 6, Father). Yet, across the sample, children drew more heavily on the mother not only for home-school support but also for directions on what to do with their free time.

Notwithstanding these difficulties, working from home offered parents more flexibility in combining work, family life, and household chores. Shifting her teaching work online, one mother managed to increase the number and geographic range of participants, and to combine work with taking her son on a holiday; working from home allowed a father to spend more time with his family and to share household chores and childcare. Conversely, some mothers were frustrated about COVID-19 thwarting plans to restart work after a period of parental leave, not being able to increase their working hours as planned, and/or having to neglect their own career because of family responsibilities.

So, um, yeah, so then I was trying to work nearly fulltime with the baby and two kids at home, and it was horrible. […] Yeah. Um. I tried to keep up the normal routine […] but it just didn’t work because […] they needed help, just physical help moving around the platforms and all of those things. Um. so yeah, I was just interrupted constantly and then, um, I basically did my work at night once the kids had gone to sleep […] and yeah, I think, I think it just, it was just all of a sudden, you know, we have roles, don’t we? And my role as a mom is very different to that of a teacher. (Family 2, Mother)

Parents initially welcomed school re-openings. Yet, when schooling was only offered for a few hours a day, sometimes days apart, with different schedules for each child, parents considered it an additional burden. A child, by contrast, enjoyed alternate schooling, “because one week I am at home relaxed, can do things in peace and one week, then, with help from the school. That was somehow the best for me.” (Family 5, Daughter, 14 yrs). Children tended not to reflect on parents’ additional stress of alternate schooling, yet were aware of mothers being strained: “I also noticed that my mum was a bit cheesed off because I was home the whole time” (Family 6, Son, 9 yrs).

##### COVID-19 buttressing gender roles

While children focused primarily on their own experience of COVID-19 and home-schooling challenges in the interviews, they acknowledged the mother’s role more readily than the father’s in helping them with schoolwork and being their prime addressee for concerns.

I also thought it was a bit, not nice for my mum because she had to look after [name of younger brother] *and* my brother *and* me and to cook and to watch out for the baby and to bring him to bed and she always had to help me, if I needed help, and my dad was working all the time. It was like my mum was doing all the work then, yeah. (Family 2, Daughter, 9 yrs)

The son of the same family depicted the father as mainly unavailable due to work but occasionally helping him with homework, whereas the mother was also the playmate and idea-giver.

And, um, when I had COVID-19, I always, my father taught me how to multiply and divide. […] So, so, my father tended to work more, and my mother looked after [name of little brother] and […] my mother sometimes played with me, she also, sometimes, um, also, gave me an idea. (Family 2, Son, 8 yrs)

In two families, interviewees stressed that the father was mainly absent or working in a different room away from the rest of the family who had to share the living space for office- or home-work, sparking further considerations of gender inequity.

So, we ended up the three months, me being here with the three kids in different like home-schooling ways like tablet, computer, work and so, and my husband being in our bedroom for work. We used to exchange when I was teaching but mainly, I was here with the kids and he was there […] I think it came quite quickly back to normal of my husband working in his office, and me being with the kids. It didn’t make such a big difference. And, I think, it even accentuated differences again between men and women. I could see all my colleagues. I mean, I am working mainly with women […], and they were all in the kitchen, in the living room with one kid to the left, one kid to the right, so I know the kids of all my colleagues because they showed up at some point in the conference, yeah (laughing) so I think, you could see the inequity of it everywhere. […] My husband is cooking, he is doing things, it is not like that he is doing nothing, still the main time in the family was like, yeah, it just amplified an existing situation, it didn’t create it. (Family 3, Mother)

While fathers also described their struggles of combining work and family life, they acknowledged the primary burden being placed on the mother. “And my wife has actually carried the main burden as far as family life is concerned, with looking after homework, uh, cooking at lunchtime (clears throat)” (Family 6, Father). Relief from child-care responsibilities came through the mother-in-law for family 6, giving the father time to work, the mother time away from the family, and the children time with the grandmother, yet outside help was not an option during COVID-19 for most families.

Family 7 was the only one that described a reversal of roles: the father was at home looking after the child, while the mother was working externally. The mother perceived her husband as struggling at times, while the father did not see any “major differences” in his role before or during COVID-19 (Family 7, Father). Yet, both parents mentioned the child’s access to emergency schooling in the interviews, which gave the father time for his studies. While the father engaged in a caring role and also cooked for the child, the child sat on the mother’s lap during the interview underscoring her role as carer.

##### Refocusing on the nuclear family

While some participants, irrespective of age, reported no change in their family relationships, the dominant view was that spending more time together had strengthened family ties. Some parents recalled newly established social activities such as watching news programs for children or films together, sharing more personal experiences within the family, undertaking “micro-adventures” (Family 1, Mother), going for walks together, or other joint activities.

I would say the first lock-down, yes, because we were like really sort of like in a bubble like not meeting anyone and so, so it was like really time together and trying to do fun things together and cooking, baking, gardening […] so, yeah, we did different things that we don’t do usually, so that was nice. (Family 3, Mother)

Children viewed having their parent(s) at home as “nice” (Family 2, Son, 8 yrs) and spending time with the family as “even fun” (Family 9, daughter 14 yrs), yet joint activities mentioned by mothers such as gardening and cooking were not reflected in children’s narratives. Children talked about watching films together, going for walks, playing games, talking more with parents, and growing closer as a family, but also viewed prolonged exposure to the nuclear family as leading to conflict: “we quarrelled because we spent too much time together” (Family 6, Son, 9 yrs). Perceptions could, however, vary within the family. In Family 6, father and son described a deteriorating family atmosphere due to COVID-19.

At some point I lose my nerves and my child has, or my children have had enough of always seeing the same person. […] the mood … was often tense, because everyone was under pressure all day. Um, that was very unpleasant, especially over a longer period of time. […] The longer the lockdown, the longer at home, the more tense it was. (Family 6, Father)

Conversely, the mother noticed no changes in family relationships. “That was just a tough time that one had to go through. But we don’t have, I don’t think, a, a different relationship with each other now, with or without COVID-19” (Family 6, Mother). Differing parental perceptions could thus prevail.

In one family, the mother recounted that her husband moved back to their home-country for lack of a professional perspective during COVID-19 and that they got divorced which she depicted as not only the result of the pandemic situation. The daughters made no mention of the divorce: “And the relationship with my mother and my father were kind of, well, I didn’t really feel a change” (Family 5, Daughter, 14 yrs). The younger daughter compared her good relationship with her sister to the one between her mother and father. Only at the end of the interview did the older daughter mention visiting the father; her younger sister seemed to ignore the father’s absence showing a marked difference in how a parent and the children spoke or kept silent about an incisive family event.

#### Coping with social expectations and demands

Imposed precautionary measures affected the entire family, yet accounts varied between parents and children, especially regarding socially and psychologically challenging experiences.

##### Dealing with perceived COVID-19-related stigma

In family 1, the parents highlighted the experience of being treated differently due to COVID-19: the father was seen as a potential spreader because of his dental profession. “So, I (laughs), when they [the regulations] relaxed, my, my favourite sister-in-law, she greeted me. “Ah, come, let me hug you anyway, even if you are a ‘hot spreader’” (laughs) […] In the beginning, many people stigmatized others” (Family 1, Father). He perceived his children tensing up when testing and recounted that the daughter had been sent home because of a false positive test result. The mother shared that the son became the first local COVID-19 case: “It was discovered in [name of son] through a test in [school]… we were, I think, like the first family here that had COVID-19 then […] so, the kids sometimes heard a little bit of ‘you are to blame’” (Family 1, Mother). The son, by contrast, even when probed about the experience of testing positive to COVID-19, recounted how classmates brought him homework and that he had little to do for school when he was infected. The only negative experience was his brother’s reaction who “annoyed” him by “permanently wearing a mask” on the first day of his infection (Family 1, Son, 10 yrs). He also preferred testing for COVID-19 before going to school to have more leisure time while the others had to do COVID-19 tests in school which “was somehow funny” (Family 1, Son, 10 yrs). Parents’ concerns about stigma were thus not reflected in the child’s accounts.

Parents perceived their children across all age groups as quickly adapting to the testing requirements after initial uncertainties. “How does it work? How deep [does it have to be inserted] in the nose? […] and then they [the children] got used to it” (Family 5, Mother). This even held true for very young children. “I mean, […] they are able to do this with both hands tied. Even these (points to 5-year-old twins) test. That was the least problem” (Family 6, Mother). While parents emphasized testing as a new routine, children provided more details about their testing experience. Some compared the different test types and declared their preference for tests that were less embarrassing such as the ‘lollipop-test’.

because… the one with the spittle, the spittle usually dripped out of the pipet onto the table and, um, the one with the nose, that is okay, too, but… I don’t think, the, the one in the nose is so, well, it tickles. (Family 6, Son, 9 yrs)

For some children, reservations remained about the testing procedure, depicting themselves as “being afraid” and uncertain of the outcome. A child underscored the issue of embarrassment of being seen by others in the class while testing: “I don’t like it when you have to insert this and then everyone watches you, and my friends annoyed me with it” (Family 8, Daughter, 14 yrs). Conversely, her sister reported enjoying the time of testing at school: “We had fun when we tested, because some had to sneeze in such a funny way. And we had fun because we were able to see each other’s faces” (Family 8, Daughter, 13 yrs). For children, the testing experience thus differed considerably, ranging from a continued fear of testing positive, detesting the testing procedure or feeling embarrassed about it to enjoying testing as a way to see classmates without a mask.

##### Re-socialising after COVID-19

Some parents critically remarked that children spent too much time online and were too energetic to be indoors. With schools re-opening, social skills had to be re-learnt. In family 6, the parents’ and the son’s accounts highlighted movement restrictions with the son taking on the parents’ view of him becoming jittery with too little physical exercise.

He [name of son] was fit as a fiddle and wasn’t allowed out. […] I sent him to jump on the trampoline, but I can’t send him to jump, jump on the trampoline for five hours […] Then [at school], things got really rough. Well, with the boys, it also manifests itself physically. So, then they had real fights, brawls… um, I would say, to clarify the hierarchy, which could perhaps have been clarified in a softer tone one and a half year earlier. (Family 6, Mother)

While both parents drew a link between social problems and children’s isolation during lockdown, the son underscored that social problems in class predated COVID-19.

Then we couldn’t go into the [school] break anymore. That really stressed me, uh, ticked me off, and um… yes, then I was always so antsy in class because I could hardly move about… and then I didn’t pay attention very well… the classroom is already pretty full, and it’s not very big… then there was also stress between a few children several times, and sometimes, I was also a victim of bullies in the class. […]. It was like that from the beginning. Well, it’s still like that now. So, it’s definitely not because of the COVID-19 times. (Family 6, Son, 9 yrs)

The narratives thus show similarities as well as differences in *children and child* perspectives.

##### Internalizing rules

Some families highlighted the long-term psychological effects of COVID-19 on children, particularly on daughters, of becoming overly concerned with symptoms and the fear of contracting COVID-19. Hygiene rules became internalized to an extent that the mother felt medical help was needed.

We actually took her to the doctor because she would start saying things like, ehm, “I think I’ve got Corona [COVID-19]” or something about Corona, “because, um, it hurts here when I move”. And like, every tiny thing, “Oh mommy, do you think my eye looks right?” and just this constant fear … You know, those things that you want kids to be aware of but this virus thing just took over her whole well-being. Um, and if you’ve got an 8-year-old saying to you ten times a day “oh, ehm, do you think, I’ve washed my hands properly?” […] And she would be washing them ten times at school. It sounds like she’s got some really weird disorder, and she hasn’t. It was just that she was scared! […] And so, the doctor did like a full check and said, “Look, [name of daughter], I think everything is fine. You are like the fittest lit (not fully pronouncing little) girl I have ever seen […] There is nothing wrong with your lungs. You haven’t got Corona”. And I am very grateful for her to do that because sometimes moms saying that it’s all okay isn’t enough. (Family 2, Mother)

The daughter’s narrative tallied with the mother’s in depicting the daughter’s deepfelt concern about COVID-19. Yet, the daughter highlighted her conscious efforts to restrain herself from worrying.

Now, I feel a lot safer because I have had (pauses, searches for right word) vaccinations, and now like everyone’s safe. And I have already had Corona [COVID-19], and it wasn’t as bad. […] I normally said to myself: “Don’t think about it” – because then it’s a waste of your life when you keep thinking about a deadly bug. And then I did start stop thinking about it but I also sometimes think about it. I would think about the bug, yeah (whinerly voice) (Family 2, Daughter, 9 yrs).

Both mother and daughter recounted how the detrimental psychological effects of COVID-19 were gradually subsiding with the mother highlighting parental and general practitioner support, the daughter underscoring the absence of negative personal experiences of COVID-19 and the protection through vaccination.

Parents further recounted internalizing COVID-19 rules, sometimes in contrast to people from their home-country: “they consider us as crazy Germans, we were too careful and wanted to keep masks and meet people outside” (Family 3, Mother). Her 7-year-old daughter spoke about rebuking strangers who did not wear the face-mask properly.

The internalization of the COVID-19 precautions led to strictly obeying the rules in some families: “Me and my children, we follow the protocol. When Germany says ‘use mask’, we use mask. When Germany says ‘be at home’, we be at home” (Family 9, Mother). Considerations regarding migrant status may have reinforced this tendency, yet children irrespective of migrant status upheld the regulations: “We have to abide by the rules and wear masks again, otherwise it’s not healthy for us. That’s why it’s better when mandatory masks return” (Family 8, Daughter, 13 yrs). In a non-migrant family, a father critically commented on his daughter’s reticence to go into cafés when someone was sitting at each table, even if the café was not full, imitating her voice: “It’s too crowded. There’s no way we’re going in there” (Family 1, Father).

##### Communicating in the vernacular

Parents argued that schooling during COVID-19 focused too much on core subjects, with children missing out on music, sport, and other subjects; yet, with the exception of one father from West Asia, they hardly raised concerns about their children’s general future prospects. Mastering foreign languages online was, however, regarded as an additional burden for children. As the majority of the interviewees came from bilingual families, the acquisition of the German language was a concern for a number of parents who feared that prolonged closure of schools would have a detrimental effect on learning and improving German. A mother mentioned her son’s stutter and poor German when he was in kindergarten.

[My son’s] friends in the kindergarten weren’t German; his German wasn’t good enough… So, and he gets to school and all of a sudden, he is surrounded by five, basically five lovely full-blood German boys that speak perfect German. And I would say within like, I don’t know, yeah, the six weeks before lockdown happened again, he just flipped like that, just totally changed to it almost being his preferred language […] it felt like we just crossed over a bridge, and he was where he was supposed to be emotionally, his stutter stopped and then… lockdown… And he wasn’t surrounded by those five boys that brought him out of whatever it was. (Family 2, Mother)

The son did not talk about language issues but clearly preferred to have the interview in German, which he spoke fluently. Overall, children did not mention language-related fears; one child remarked upon her improved German which she related to physically attending school during COVID-19. “I was actually in like, I was still learning German that time when Corona began… They tell me that I could go to class because it’s okay. My Deutsch is a little bit better” (Family 9, Daughter, 14 yrs). Children in this study recounted mixed experiences with online teaching [45] from enjoying lessons: “I like German more than maths. On zoom we do more German” (Family 4, Son, 8 yrs) to losing heart: “and then I also learned less, somehow understood less, with the online tests it was also difficult […] My motivation went away like that, that was indeed stressful” (Family 5, Daughter, 14 yrs).

#### Re-evaluating restrictions and life’s priorities

Precautionary measures led to some parental re-evaluation of COVID-19 restrictions and priorities in life. Parents tended to reflect on the effects of the restrictions on schooling and leisure activities on children’s lives; they also self-critically questioned the leisure activities program they had imposed on the children before the pandemic. Children tended to describe the measures and their experiences; the wider societal picture was hardly mentioned.

##### Highlighting social and age-related injustices

A number of parents questioned the proportionality and consistency of precautionary measures particularly as they affected children. “What is the actual risk [of certain activities]? […] I think, the proportion was missing” (Family 1, Mother). They underscored different sets of rules for different groups: “like, oh, you can be outside with people and you could be outside like in a bar drinking but my kids weren’t allowed in school” (Family 2, Mother). Children and families having to pay the price for other societal groups like the elderly or at-risk-patients was raised in some interviews. “Somehow we had the impression that kids are locked out or families in general, and nobody paid attention to the, the needs of children” (Family 1, Mother).

##### Re-evaluating leisure activities

Leisure activities, places, and meeting points such as playgrounds, sports clubs, music lessons, horse-riding, or dancing formed an important offset to schoolwork; discontinuation of activities and closure of sports venues was remarked upon negatively across families as restricting children’s need for physical activity.

…and no more sports for [name of son], no swimming all of a sudden, no swimming lessons, no… uh, no… Taekwondo, no piano lessons anymore, five months absolutely nothing for him, just staying at home […] In the beginning we were only at home because they closed all the playgrounds, unfortunately (Family 4, Mother).

In a similar vein, the son highlighted that he preferred the time before COVID-19, since he was able to meet his friend and “mostly went to the playgrounds” (Family 4, Son, 8 yrs).

The restrictions, however, also triggered a reflection on the many organized leisure activities parents had imposed on their children: “Life in general is too fast and we do too many things, and we meet too many people” (Family 3, Mother). More free time for the children could lead to developing new interests.

It made me realize how much I get my kids to do every day and that it is not necessary all the time, and all of these activities […] it really occurred to me, that I pile on too many activities because what she [daughter] would do is all of a sudden, she developed this love of reading. (Family 2, Mother)

Children spoke generally of their hobbies and new online activities and some commented on the strangeness of doing music lessons online. For some, COVID-19 also provided the opportunity to discontinue activities they were not enjoying such as private music lessons or sports activities: “it was not so bad, and I was not opposed to the fact that they [swimming lessons] seized for a while” (Family 5, Daughter, 14 yrs). Thus, COVID-19 could also have a positive effect on their leisure time.

##### Reflecting on privileges

Despite facing difficulties, many parents spoke of their privilege of having a garden or a forest nearby, of being able to work and look after the children: “I mean, we were lucky […] but some kids were alone the whole day” (Family 3, Mother). Also, some counted themselves privileged in having a partner and not being confronted with serious social challenges:

Oh, yes, also, single parents who live in a small flat, they really have it unbelievably much more difficult than us here. That would be horror, if I simply had to, had to go through that, absolutely […] Well, if someone is also violent or has a child who might have ADHD or something else, I think that can destroy a family very easily. Luckily, we did not have to face that. (Family 6, Father)

Emphasizing one’s own privileged situation in comparison to other families set the restrictions in context and relativized the impact. Children, by contrast, reported about their activities during and after lockdown as a matter of fact such as playing with friends or neighbours in the garden or courtyard. This was not reflected upon as a privilege.

**Fig. 1:**
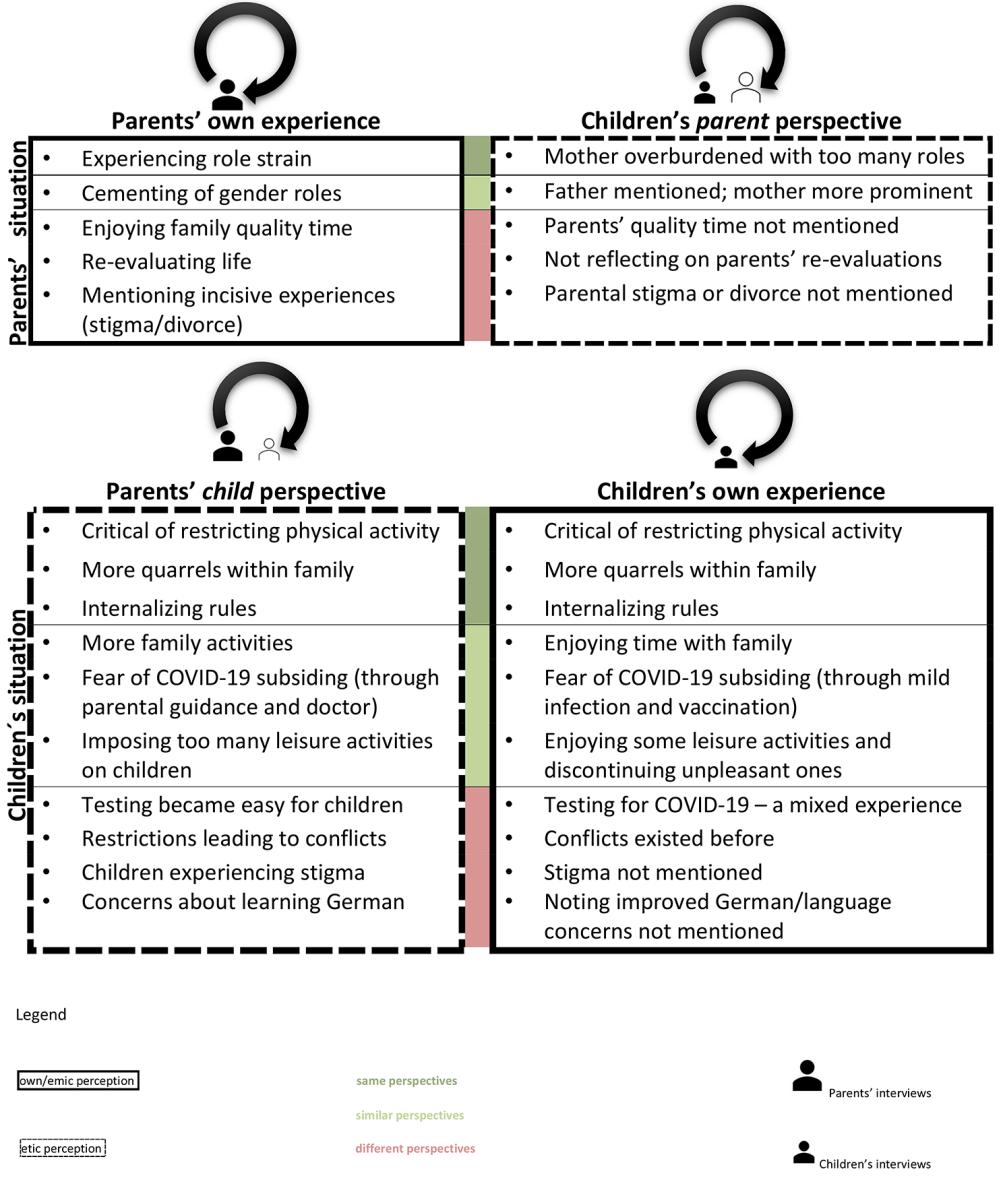
Perceptions by parents and children

In summary, while parents and children highlighted similar sentiments with regard to some of the COVID-19 restrictions, the views particularly on incisive experiences could vary considerably across the accounts of family members. Figure 1 illustrates the different perspectives portrayed in this article, their overlap and divergence: *Child* perspectives (etic) vs. children’s own experiences (emic) and parents’ own experiences (emic) vs. what we termed *parent* perspectives (etic).

## Discussion

Interviews with parents and children revealed a nuanced picture of their attitudes and experiences of COVID-19, including temporary confinement to the nuclear family, facing physical activity restrictions, psycho-social issues such as COVID-19 fears, internalized rules, stigma, a range of testing experiences, and a re-evaluation of life’s priorities. Despite exposure to the same precautionary measures, the salience of issues varied between parents and children within and across families, from being a non-issue for children to diverging and partly overlapping accounts of parents and children.

Our application of emic and etic in contrasting family members’ own versus other family members’ perspectives moves beyond the classic understandings of this paradigm. Pike [15] would have likely viewed parents and children as being inside the family “system” and thus belonging to the emic dimension. We applied emic only to interviewees’ own views/experiences, and etic to family members’ views of the respective other, thereby drawing on Pike’s notions of differing standpoints that may “shade into another” [15]. This nuanced insider and outsider perspective revealed that despite family members knowing a lot about each other, their perspectives – especially the parents’ perspective of children and children’s own perspectives – were not necessarily congruent. Also, parents’ accounts often provided more context to their children’s accounts.

The emic/etic framework adds another dimension to sociological studies of children and childhood. Going beyond parents’ perspectives of children and children’s own perspectives [31, 32, 51], it also incorporates the view that children hold of their parents’ experience. Our study thus unveiled four different perspectives: parents’ and children’s own emic experiences and views, and the etic perspectives of the two groups (a *child* perspective, i.e., parents’ views on children’s experiences, and a *parent* perspective, i.e., children’s views on parents’ experiences). This *parent* perspective is, to the best of our knowledge, rarely found in qualitative studies with children. Quotes in studies with children highlight children’s own experience (a *children’s* perspective), with some showing feelings towards parents or parental behaviour towards them [31, 32]. Few studies provide quotes of children putting themselves in the shoes of their parents, and these tend to be analysed in terms of content rather than the perspective of the child on a parent [51, 52]. Further research will have to explore why a *parent* perspective seems rare in children’s interviews. While we found the *parent* perspective in 9-year-olds in our sample, it was too small a sample to draw any conclusions from it pertaining to child and adolescent developmental stages [53]. Other studies have found that girls use interpersonal narratives more than boys [54, 55]; in our study, no clear gender pattern emerged.

The *parent* perspective mainly arose in children highlighting the density of the mother’s role. As this information was shared unprompted, it can be seen as elevating the issue of role strain and the gender dimension of COVID-19, both of which were underscored by parents and children, showing an emic/etic convergence of views. While fathers highlighted their own juggling of roles, overall, a father’s role strain was not as readily acknowledged by other family members in our study and thus showed emic/etic divergence. Being at home provided the opportunity for fathers to share household chores, take on home schooling tasks and play more with their children, thus mirroring findings from an Australian study [56]. Mothers and children in our study, however, depicted the mother as central for leisure activities during lockdowns and home schooling. The absence or non-availability of some fathers, as described by some mothers and children, further intensified the mothers’ role strain and overburdening. This aligns with an Italian study on parents working in academic jobs [57], and a British study on the increased workload of women and difficult reconciliation of working from home with family duties [11]. A re-emergence of gendered household roles as part of COVID-19 restrictions has also been found in gender-progressive countries like Iceland or Norway [58, 59]. In future epidemics, policy-makers should ensure that particularly mothers do not become overburdened with too many additional concurrent tasks placed upon them through precautionary measures.

Parents’ *child* views and children’s own accounts overlapped across families in cherishing joint family time and activities during COVID-19 as well as noting more quarrels and tension. Our study also showed differing perceptions of conflict among parents in the same family highlighting in-group variation. This band-width of family experiences has been named the “push-pull of intimacy” in an Australian study [60].

Interviewees’ disappointment across our dataset to see public sports facilities closed during COVID-19 echoes findings of a Canadian study [61]. Parents’ views of privilege in having a garden or forest nearby and children’s matter of fact description of playing sports in the garden in our study resonate with a US-American study highlighting greater physical activity levels of children in homes with a yard, garden, nearby woods, or open spaces [62]. The role of the socio-economic status of parents and the location of the parental home impacting on children’s opportunities for physical activities during the pandemic [63, 64] was, however, less obvious in our study. While a garden was the prerogative of richer families, parents living in more confined spaces spoke about going for walks with their children.

Parents’ etic views of children’s experiences could diverge from children’s accounts in our study, either in nuance or in substance. A number of mothers mentioned the psychological impact of COVID-19 on their daughters. This is also reflected in a COVID-19 systematic review which highlights the association of female gender and younger age with distress [6]. However, only one girl in our study mentioned her anxiety, and her interpretation of how she partly overcame it varied from her mother’s, showing more agency in her own account whereas the mother’s account highlighted parental and doctoral guidance.

Further divergence between parents’ and children’s narratives emerged in the case of school conflicts: the parents tended to blame lost social skills during COVID-19, the son blamed the particular class composition. Furthermore, parents’ mostly positive description of child testing stood in contrast to children’s nuanced and mixed testing experience. Where parents felt that testing had become routine, some children spoke about the fear, unpleasantness, or embarrassment of testing, others of having fun. Etic and emic perceptions could thus differ considerably.

Some experiences, such as becoming stigmatized for a positive COVID-19 test or the parents’ divorce were not reflected in children’s interviews. The omission of these potentially incisive life events seemed surprising and difficult to interpret. Aged 9 to 14, the children in the respective families would have been old enough to be fully aware of the situation. Whether they did not want to talk about these issues with a stranger, pretended that everything was ‘normal’, held a different view of the matter or felt that the issue had become less relevant over time could not be fully answered.

Language acquisition emerged as a distinct etic parent concern in our sample, potentially linked to the majority of families having at least one parent with a non-German background. The literature on language-related concerns during COVID-19 is limited; a German study showed language deterioration for native and non-native school beginners during the pandemic [65]. These concerns were reflected in our study in mothers fearing that their children’s German language skills may worsen, or speech problems may re-emerge - a concern not reflected in children’s interviews.

Parents also reflected on the regulations in terms of consistency, damage to the children, and their own pre-pandemic behaviour of over-exposing children to leisure activities. Children, by contrast, focused more on describing the here and now. Particularly adolescent participants indicated that COVID-19 had presented the opportunity to rid themselves of leisure activities they were not really enjoying and shared general reflections about people’s behaviour during COVID-19. Comparisons of etic and emic value judgements of children’s experiences and quality of life within and beyond the pandemic are inconclusive [31, 37]. Strengthening creativity through reducing organized leisure activities may have also had a positive effect for some children in terms of reading and spending time with friends as depicted by parents and children. Creative leisure activities have shown to play an important role for mental health during COVID-19 [66]. The ability to make choices should, however, be provided for children and adolescents.

We note the following strengths and limitations: Our sample deliberately included many families with at least one parent who had migrated to Germany and thus our findings are specific to this sample. Language acquisition and originating from a different country with the option of returning there in case of job insecurity and a divorce show migration-specificity. The average educational qualifications of parents in our sample was high, and all children seemed to have many leisure activities irrespective of migrant background, which might reflect the desire to provide the children with as many opportunities as possible. COVID-19 publications based on quantitative studies have noted high rates of psychological distress among migrants [5, 6] and migrant adolescents [67]. In our study, some migrant families had access to emergency schooling during COVID-19 which may have eased the social and learning situation.

Additionally, while we probed adult participants on their view of how children had experienced COVID-19, if the parents did not already mention it in the interview, we asked children generally about their perceptions of family life during the lockdown, and not explicitly about their parents’ experience. Children’s unprompted comments about parents’ role strain thus could be seen as highlighting an elevated importance of the topic.

## Conclusion

Juxtaposing parents’ and children’s perspectives adds nuance to the literature on how families managed life during the pandemic. Including the perspectives of migrant families should be part and parcel of research within and outside epidemics. Future prevention and mitigation efforts in pandemics should also consider their psycho-social effects on parents and children. Parents’ wish for less overburdening and for more reliability and consistency of precautionary measures in order to combine work and family life and children’s wish for more physical activities, leisure activities of their choice, socializing and open schools should inform measures in future health emergencies. However, parents’ re-evaluation of life’s priorities and children’s discontinuation of unfavoured leisure activities also underscored COVID-19 as a catalyst for changes that family members perceived as positive.

While emic and etic have been mainly applied to the researcher’s stance [16, 68], we have employed it in family research in what could be regarded as an emic realm – gaining insight into family members’ experience and views by interviewing them. The outsider here is not the researcher but another family member who provides his or her etic view of the other family member’s emic experience. By interviewing parents, we gained deeper insights into their own perceived role strain and gendered roles, and of their etic view of children’s psychosocial concerns and their exposure to stigma; by interviewing children, we captured the richness and variety of their emic experiences and empathetic etic view of the mother’s role. Combining these perspectives provided deeper insights into family members’ navigation, experience and views of COVID-19 measures. We recommend to further explore convergences and divergences of emic and etic family perspectives in order to obtain a more comprehensive picture of family members’ experience and response to precautionary epidemic measures. This will provide valuable insights for future pandemic guidance.

## Data Availability

According to the study protocol, the interview data can only be made available on reasonable request to researchers conducting research related to children and COVID-19.

## Footnotes

1
https://www.destatis.de/EN/Themes/Society-Environment/Population/Migration-Integration/Tables/foreign-population-laender.html - accessed 19.4.2024.

2
https://www.gew-bw.de/aktuelles/detailseite/corona-rueckblick-2020 - accessed 19.4.2024.

3 We will use the term “children” generically unless the age-group is relevant.
